# Clinical Biomarkers and Pathogenic-Related Cytokines in Rheumatoid Arthritis

**DOI:** 10.1155/2014/698192

**Published:** 2014-08-21

**Authors:** Xiaoyin Niu, Guangjie Chen

**Affiliations:** Department of Immunology and Microbiology, Shanghai Jiao Tong University School of Medicine, Shanghai Institute of Immunology, 280 South Chongqing Road, Shanghai 200025, China

## Abstract

Rheumatoid arthritis (RA) is a common autoimmune disease with unknown etiology and pathogenesis. Although major therapeutic advances have been made in recent years, there is no cure for the disease. Current medications mainly reduce inflammation in order to relieve pain and slow joint damage, but many have potentially serious side effects. Therefore, to find specific biomarkers will benefit both RA patients to find relief from the disease and physicians to monitor the disease development. A number of biomarkers have been discovered and used clinically, and others are still under investigation. The autoantibodies, which are widely used in diagnosis and prognosis, novel biomarkers, which reflect clinical disease activity, and newly found biomarkers and pathogenic-related cytokines are discussed in this review.

## 1. Introduction

Rheumatoid arthritis (RA) is a chronic inflammatory disease of polyarticular arthritis affecting approximately 1% of adults worldwide [1, 2]. It typically leads to deformity and destruction of the joints and systemic disorders throughout the body as well. Although the etiology and pathogenesis of RA remain unknown, immunological hyperreactivity caused by large numbers of T cells, mostly CD4, and plasma cells is generally considered to be important to contribute its development. The clinical diagnosis of RA is based on several criteria, including physical symptoms, joint radiographs, and serological tests [3]. In the ACR/EULAR 2010 classification criteria, “definite RA” is defined based on the confirmed presence of synovitis in at least 1 joint, absence of an alternative diagnosis that better explains the synovitis, and achievement of a total score of 6 or greater (of a possible 10) from the individual scores in 4 domains: number and site of involved joints (score range 0–5), serologic abnormality (score range 0–3), elevated acute-phase response (score range 0-1), and symptom duration (2 levels; range 0-1) [3].

Multiple studies have revealed RA-related autoantibodies and numerous biomarkers including cytokines/chemokines as well as erythrocyte sedimentation rate (ESR) and C-reactive protein (CRP) orchestrate pathological processes in RA [4–6]. During the course of the disease, RA patients need to be diagnosed very early, possibly before diagnostic criteria are fulfilled or maybe even before clinical symptoms are apparent. Early identification of patients with RA will help improve clinical outcomes with early treatments. Then, markers of disease activity and severity are needed. Finally, screen tests for prediction of response to therapy and progression of the diseases are also necessary.

A biomarker, also known as biological marker, generally refers to a measured characteristic which may be used as an indicator of some biological state or condition. This term occasionally refers to a substance whose presence indicates the existence of living organisms. Biomarkers play pivotal roles in disease diagnosis and interventions at early stage and are also helpful in knowing the state of treatment and how body is acting or responding to the medication. Therefore, exploring and measuring biologic markers in blood or in joint fluids may serve as not only indicators of diagnosis but also indicators of prognosis and the subsequent response to therapy. A good biomarker can be used to measure the disease progress and the treatment effectiveness. It is a parameter which can be chemical, physical, or biological. Here, we discuss the biomarkers and pathogenic-related cytokines involved in clinic, pathogenesis, and prospection in RA.

## 2. Clinical Biomarkers

The biomarkers currently used for the diagnosis of RA are mostly clinical. Now there are several useful clinical biomarkers, including various autoantibodies, such as rheumatoid factors (RF), anti-perinuclear factor (APF), anti-keratin antibodies (AKA), anti-filaggrin antibodies (AFA), and anti-cyclic citrullinated peptide antibodies (anti-CCP). Erythrocyte sedimentation rate (ESR) and C-reactive protein (CRP) also have associations with RA [7].

### 2.1. Autoantibodies as Clinical Biomarkers

Rheumatoid factor (RF), an antibody to the IgG Fc region, is a classic feature of the disease with sensitivity of 60–80% [8], but its specificity is not very high since it can also be detected in other autoimmune diseases, such as systemic lupus erythematosus (SLE) and Sjogren's syndrome (SS). The key pathogenic markers are IgM and IgA rheumatoid factors in RA [2]. Showing perinuclear fluorescence, APF is probably an antibody of the 7S gammaglobulin type against keratohyalin granules in buccal mucosa cells and is high in RA patients compared to the healthy people [9]. AKA, a naturally occurring antibody that reacts with the keratinized tissue, is found in the serum of around 60% RA patients but not in healthy people [10].

Autoantibodies induced by citrullination, conversion of peptidyl-arginine to peptidyl-citrulline, can be measured efficiently by using cyclic citrullinated peptides (CCP) as antigens [11, 12]. The detection of anti-CCP antibodies, as well as RFs, particularly IgA-RF, has a strong predictive value for diagnosis in early RA [12, 13]. Not only ESR but also anti-CCP is an important predictor of early bone mineral density loss [14].

Similar to RF, antibodies against citrullinated proteins (ACPA) have correlation with bone destruction, but the latter is associated with larger bone erosions by directly initiating the differentiation of bone-resorbing osteoclasts [15, 16]. Moreover, the bone loss caused by ACPA occurs before disease onset in ACPA-positive patients [17, 18]. The knowledge on ACPA made great advancement in the field of RA in the past decade and this biomarker has already been included into the ACR/EULAR 2010 diagnostic criteria. Using both RF and ACPA may be helpful in diagnosis and classification of RA [19, 20]. Furthermore, anti-mutated citrullinated vimentin (MCV) antibodies have been reported as a fairly sensitive serological marker of RA and were significantly higher in early RA patients [21].

### 2.2. CRP and ESR

In clinical management of RA, CRP and ESR are commonly ordered tests to guide diagnosis of RA besides the measurements of the mentioned autoantibodies. CRP is a protein, the levels of which rise in response to inflammation. Increased CRP was detected before the onset of RA [22] and also indicated the activity of the disease during the course of the disease. Erythrocyte sedimentation rate (ESR) is a nonspecific measure of inflammation and can be useful not only in diagnosing autoimmune diseases, such as rheumatoid arthritis, but also in monitoring the disease process. It can be an index observing the activity and severity of RA synovitis. The combination of ESR and CRP may improve sensitivity and specificity of the diagnosis of RA [23, 24].

### 2.3. Newly Found Clinical Biomarkers

What is more, levels of serum calreticulin (CRT) are now detected to be increased in patients with RA compared with those controls and have a significant correlation with disease activity in RA. It might be a potential clinical biomarker [25]. Likewise, there is an autoantibody system that discriminates between citrulline- and homocitrulline-containing antigens in the sera of RA patients. Anti-carbamylated protein (anti-CarP) antibodies IgG and IgA were observed in RA sera [26]. Interestingly, these anti-CarP antibodies are also present in around 20% of the ACPA-negative RA patients and have association with more severe joint damage, indicating they could serve as a unique and relevant serological marker for ACPA-negative RA [27].

Important associations between the above clinical markers and severity of RA have been noted and used in aiding diagnosis of RA, monitoring disease progress, and assessing prognostic in patients with established disease.

## 3. Investigational Biomarkers

**Figure 1:**
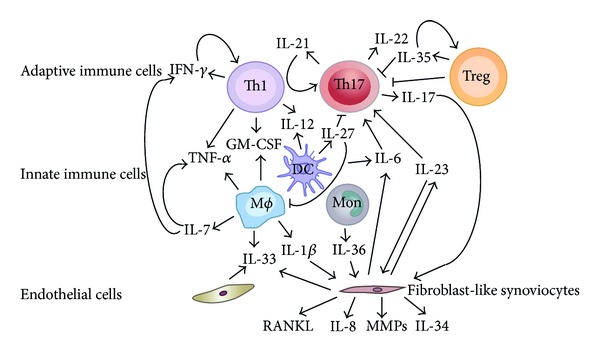
Network of investigational and prospective biomarkers in RA. A variety of different cell populations, including adaptive immune cells, innate immune cells, fibroblast-like synoviocytes, and endothelial cells, secrete cytokines as biomarkers not only in serum but also in synovial fluid and orchestrate inflammation and bone destruction in RA patients.

Besides the clinically used biomarkers, scientists have been engaging in investigating potential biologic markers for many years. Cytokines serve as molecular messengers between cells and there are multiple studies revealing that numbers of cytokines have been involved in the pathogenesis of RA by triggering or regulating the inflammatory responses (Figure 1) [28, 29]. Overexpressions of certain cytokines, such as IL-1, IL-6, IL-8, IL-17, IL-21, tumor necrosis factor (TNF)-*α* and granulocyte-macrophage colony-stimulating factor (GM-CSF), were observed in RA patients [30–35]. These cytokines could promote synovial membrane inflammation and osteocartilaginous resorption via stimulation of osteoclastic mediators [35].

### 3.1. Th1, Th17 Cells and Related Cytokines

Autoreactive T cells, such as Th1 and Th17 cells, are thought to play important roles in autoimmune pathology of RA [36–38]. The percentage of interferon (IFN)-*γ* expressing cells was slightly increased in peripheral blood mononuclear cells (PBMC) but highly increased in synovial fluid mononuclear cell (SFMC) of RA patients [31]. IL-12, a Th1 cytokine, was increased in serum and synovial fluid in RA and its level correlated with disease activity score [39].

The discovery of Th17 cells enriched our knowledge and understanding of RA pathogenesis. Th17 profile therefore has shown pathogenic role in RA [40–42]. Increased IL-17 was reported in the sera and synovial fluid of RA patients [42–45]. Neutralizing IL-17 treatment reduced the severity and slowed the progression of collagen-induced arthritis (CIA), a mouse model of human rheumatoid arthritis [46]. Secreted by Th17, IL-21 and IL-22 were also observed at a high level in RA patients compared to the healthy controls or osteoarthritis (OA) patients [43, 47]. In addition, IL-21 autoregulated its own production in human CD4+ T cells and enhanced Th17 proliferation, leading to the expression of RORC production, and was strongly associated with the levels of auto-antibodies and disease severity as well [31, 48]. IL-23 is known to promote IL-17 production [49] and was detected in RA synovial fibroblasts, but, like a loop, it can be upregulated by IL-17 in RA synovial fibroblasts via PI3-kinase/Akt-, NF-*κ*B-, and p38-MAPK-mediated pathways [50]. IL-23 directly induced osteoclast differentiation by upregulation of receptor activator of NF-*κ*B (RANK) in mouse myeloid precursor cells and RANK ligand (RANKL) in human fibroblast-like synoviocytes [51, 52]. RANK/RNAKL signaling regulates the formation of multinucleated osteoclasts from their precursors as well as their activation and survival in normal bone remodeling and in a variety of pathologic conditions [53]. In RA synovial tissue, p19 subunit and RANKL have positive correlation and might contribute to bone destruction in RA [54, 55].

### 3.2. Other Proinflammatory Cytokines

IL-1 is produced by a variety of cells that are part of the innate system and mediates bone resorption and cartilage destruction. The IL-1*β*-NF-*κ*B axis is central in the production of proinflammatory mediators in the inflamed synovium [56, 57]. NF-*κ*B activation by IL-1*β* induced the gene expressions of matrix metalloproteinases (MMPs) that are major products of cytokine stimulated fibroblast-like synovial cells (FLS) and efficiently degrade the collagenous components of cartilage and bone, leading to the joint deformity and pain in the patient with RA [58]. Additionally, proinflammatory cytokine, IL-6, has important effects on the differentiation and activation of B and T cells, macrophages, osteoclasts, chondrocytes, and endothelial cells and broad effects on hematopoiesis in the bone marrow [35].

Together with TGF-*β*, IL-6 is the key inducer of Th17 via signal transducer and activation of transcription-3 (STAT-3) [59] and its level was significantly associated with clinical symptoms and levels of clinical biomarkers [60].

Additionally, TNF-*α* has shown its contribution to the pathogenesis of RA at early stage. It is produced locally in the joint by synovial macrophages and lymphocytes infiltrating the joint synovium. TNF has been recognized as a key pathogenic cytokine that drives a pathogenic cytokine milieu leading to tissue damage [61, 62].

### 3.3. Newly Emerged Cytokines/Chemokines

Other cytokines/chemokines, such as IL-8, IL-9, IL-15, leukemia inhibitory factor (LIF), granulocyte colony-stimulating factor (G-CSF), chemoattractant protein-1 (MCP-1), growth-related oncogenes GRO-*α* and GRO-*β*, CCL19, CXCL12, and CXCL13, were upregulated in preclinical or clinical period of RA development by induction of IL-17 or TNF-*α* and IL-1 synergically [4, 5, 63, 64].

Therapeutic strategies that specifically block these investigational inflammatory cytokines are expected to be highly effective in treating RA patients. Actually, several immunotherapies have been developed and tested in RA treatment. The biological therapeutics include TNF inhibitors, IL-1 inhibitors, IL-6 inhibitors, and IL-17 inhibitors, as well as B cell depletion, costimulatory blockers, and JAK inhibitors [20, 35, 65]. On one hand these approaches neutralize the inflammatory mediators and show effectiveness in clinical trial; on the other hand, their effect can be evaluated by measuring the concentration of the inflammatory cytokines combined with clinical biomarkers, such as RF, ACPA, ESR, and CRP.

## 4. Prospective Biomarkers

Recently, several interleukins involved in inflammatory joint diseases have been identified and characterized [66] (Figure 1). These new biomarkers could give us more understanding of RA pathogenesis.

### 4.1. Pathogenic Cytokines

IL-7, which is an IL-2 cytokine family member, involves T cell-driven autoimmunity, inflammation, and tissue destruction [67, 68]. The abundant intra-articular expression of IL-7 was detected in RA synovial fluid [69]. In addition, IL-7 and IL-7R were coexpressed on RA synovial tissue lining and sublining macrophages and endothelial cells [70]. This cytokine can stimulate TNF-*α* and IFN-*γ* production and its level correlates with disease activity, suggesting it has value as a diagnostic biomarker predicting the progression to RA [71, 72].

The overproduction of IL-33, a newly identified inflammatory cytokine, was found in synovial fluids of RA patients. It could be upregulated by hypoxia-inducible factor-1*α* (HIF-1*α*) which was also increased in RA synovium through the activation of p38 and ERK pathways. And IL-33 in turn induced more HIF-1*α* in RA. These two participants exacerbate the disease severity and may serve as new therapeutic targets in biologic treatment [73].

IL-34, expressed by giant cell tumours of bone, plays a critical role in macrophage differentiation and osteoclastogenesis [74, 75]. In RA patients, it was elevated in synovium, synovial fluid (SF), and FLS. The secretion of IL-34 was upregulated in FLS by TNF-*α* or IL-1*β* induction and was mediated by the transcription factor nuclear factor *κ*B (NF-*κ*B) and activation of c-Jun N-terminal kinase (JNK), indicating its osteoclastogenic role in RA [76, 77].

Increased IL-36*α* (one form of IL-36) expression was found in RA synovium and could lead to IL-6 and IL-8 production by synovial fibroblasts through p38/NF*κ*B activation in vitro [78]. IL-36*β*, known as IL-1F8, was found to be expressed in synovial membrane in CIA and synovial membrane biopsies from RA patients [66, 79]. Another member of IL-1 family which was detected in various inflammatory diseases including RA is IL-37 which interacted intracellularly with Smad3 [66, 80].

### 4.2. Anti-Inflammatory Cytokines

In contrast to the inflammatory cytokines, IL-35 is an inhibitory cytokine that contributes to regulatory T (Treg) cells function and suppresses the pathogenic cells, such as Th1 and Th17 cells [55, 81, 82]. IL-35 belongs to IL-12 family and is mainly produced by CD4+Foxp3+Treg cells [55, 83]. It can further enhance Treg generation, suggesting that it is an autocrine cytokine [84]. IL-35 treatment could ameliorate the severity of CIA [81, 82].

IL-27 is a novel member of IL-12 family and has two sides' properties, pro- or anti-inflammatory in RA [85]. Significantly higher concentration of IL-27 was found in plasma and synovial fluid of RA patients than in OA patients [86, 87]. Expressions of ICAM-1, vascular cell adhesion molecule, inflammatory chemokines, such as IL-6, CCL2, CXCL9, CXCL10, and MMP-1, in RA FLS were induced under IL-27 stimulation [85]. Although IL-27 shows its pathogenic role, it was also found to have suppressive function in macrophage responses to TNF-*α* and IL-1*β* [88]. Furthermore, IL-27 also promotes CD4+Foxp3-T regulatory cell to produce IL-10 and downregulate IL-6 and IL-1*β* in early CIA, which resulted in suppression of Th17 and monocyte migration and vascularization [89, 90].

Although numerous studies revealed the new candidates participating in development of RA, their exact mechanisms by which these molecules modulate the proinflammatory or anti-inflammatory response still require elucidation. These emerging players not only give us new evidences in complicated pathogenesis of RA but also suggest new strategies of therapeutic intervention in RA since current biotherapies are not effective for all RA patients.

## 5. Conclusion

RA is a chronic and systemic autoimmune disease that affects many tissues and organs, especially flexible joints. It can lead to deformity and dysfunction if not timely and adequately treated. Biomarkers, not only already clinically used but also under investigation, play important roles in the development of chronic human diseases which include RA. They may serve as indicators for clinical observation on the disease progression and the therapeutic effect. Furthermore, some of the biomarkers can also be or has already been a therapeutic target for the treatment. We expect that targeting specific biomarkers with the least side effects will bring hope to RA patients.
